# Development of an estimated glomerular filtration rate formula in cats

**DOI:** 10.1111/jvim.15325

**Published:** 2018-10-30

**Authors:** Natalie C. Finch, Harriet M. Syme, Jonathan Elliott

**Affiliations:** ^1^ Bristol Renal, Bristol Medical School, University of Bristol Bristol United Kingdom; ^2^ Department of Clinical Science and Services, Royal Veterinary College London United Kingdom; ^3^ Veterinary Basic Sciences Royal Veterinary College London United Kingdom

**Keywords:** cat, feline, kidney, renal function

## Abstract

**Background:**

Estimated glomerular filtration rate (eGFR) formulas are routinely used in human patients to provide a more accurate evaluation of GFR compared to serum creatinine concentration alone. Similar formulas do not exist for cats.

**Objectives:**

To validate a prediction formula for eGFR in cats based on adjusting serum creatinine concentration.

**Animals:**

Client‐owned cats with various levels of renal function.

**Methods:**

The study was cross‐sectional. Glomerular filtration rate was determined by iohexol clearance. Variables including signalment, biochemical markers, and noninvasive measurements considered to represent surrogate markers of muscle mass were evaluated with the reciprocal of serum creatinine concentration in a multivariable regression model. The derived eGFR formula was subsequently tested in another group of cats and agreement with GFR assessed.

**Results:**

The formula was developed in 55 cats. Only a single morphometric measurement (pelvic circumference) along with the reciprocal of serum creatinine concentration (creatinine^−1^) independently predicted GFR in the final multivariate model. The derived eGFR formula was 0.408 + (243.11 × creatinine^−1^ [μmol/L]) ‐ (0.014 × pelvic circumference [cm]). When the formula was tested in another 25 cats it was not found to offer any advantage over creatinine^−1^ alone in its relationship with GFR (eGFR, *R* ^2^ = 0.44, *P* < .001 vs reciprocal of creatinine, *R* ^2^ = 0.45, *P* < .001). Furthermore, agreement between eGFR and GFR was poor.

**Conclusions and Clinical Importance:**

An eGFR formula for cats that adjusted serum creatinine concentration for a marker of muscle mass was developed. The formula did not provide a reliable estimate of GFR, and therefore, its routine use cannot be recommended.

**Abbreviations**
BCS: body condition score
BL: body length
BSA: body surface area
BW: body weight
CKD: chronic kidney disease
CKD‐EPI: chronic kidney diseases epidemiology collaboration
eGFR: estimated glomerular filtration rate
ECFV: extracellular fluid volume
FFM: fat free mass
FM: fat mass
FLH: forelimb height
GFR: glomerular filtration rate
HLH: hindlimb height
LFLC: left forelimb circumference
LHLC: left hindlimb circumference
LPHLC: left proximal hindlimb circumference
MDRD: modification of diet in renal disease
PC: pelvic circumference
RFLC: right forelimb circumference
RHLC: right hindlimb circumference
RPHLC: right proximal hindlimb circumference
TBW: total body water
TC: thoracic circumference

## 1 INTRODUCTION

Detection of chronic kidney disease (CKD) before development of azotemia in cats is desirable because implementation of therapeutic interventions at this stage may delay or prevent progression of CKD. Limitations of serum creatinine and urea concentrations and urine specific gravity for assessing renal function, particularly in early stage CKD, are recognized.^1^ Measurement of glomerular filtration rate (GFR) is considered to provide the most accurate estimation of renal function. Serum creatinine concentration is the most routinely used marker of GFR, but it not only reflects renal function but also other factors including muscle mass. Creatinine is generated endogenously from creatine and creatine phosphate in skeletal muscle cells. Therefore, methods that correct plasma creatinine concentration for a patient's muscle mass may give a more accurate estimation of GFR.

Estimated GFR (eGFR) formulas offer the advantage of more accurately reflecting actual GFR than serum creatinine concentration in human patients. By incorporating demographic and clinical variables that may affect physiological processes contributing to serum creatinine concentration, a more accurate measurement of renal function may be ascertained. Of most relevance are factors that contribute to muscle mass such as age, sex, and race. It is now mandatory for eGFR to be reported with every serum creatinine concentration measurement performed in human patients in several states in the United States, in the United Kingdom, and in Australia,^2^ highlighting the importance of such formulas. The most widely used prediction formulas for GFR in human patients are the Cockcroft‐Gault,^3^ Modification of Diet in Renal Disease (MDRD),^4^ and Chronic Kidney Disease Epidemiology Collaboration (CKD‐EPI) formulas^5^ (Table 1). These formulas estimate GFR from serum creatinine concentration by means of prediction equations that take into account of factors such as age, sex, race, and body size.^3^, ^4^, ^5^ After introduction of reporting of eGFR alongside serum creatinine concentration, recognition of CKD by doctors has increased.^6^

**Table 1:** Estimated GFR (eGFR) formulae in human patients

	Predicted clearance	Formula	Variable
Cockcroft‐gault	Creatinine clearance	[(140 ‐ age) × BW/*f*] × SCr	Age (yrs), BW; body weight (kg), SCr; serum creatinine (mg/L), *f*; factor 7.2 for males and 8.5 for females
MDRD	^125^I‐Iothalamate clearance	170 × SCr^−0.999^ × age^‐0.176^ × 0.762 (if female) × 1.18 (if black) × SUN^−0.170^ × alb^0.318^	SCr; serum creatinine (mg/dL), age (yrs), SUN; serum urea nitrogen (mg/dL), alb; serum albumin (g/dL)
CKD‐EPI	^125^I‐Iothalamate clearance	*a* × (SCr/b)^c^ × 0.993^age^	*a* is a factor based on race and sex (black women ‐ 166, black men ‐ 163, white women ‐ 144, white men ‐ 141), *b* is a factor based on sex (women ‐ 0.7, men ‐ 0.9), *c* is a factor based on sex and SCr (μmol/L; women SCr < 62 ‐ −0.329, SCr > 62 ‐ −1.209, men SCr < 80 ‐ −0.411, SCr > 80 ‐ −1.209), age (yrs)

Identifying useful methods that correct serum creatinine concentration to account for muscle mass would be important in developing an eGFR formula for cats. Methods to directly measure muscle mass cannot be readily applied to clinical patients.^7^ Skeletal muscle mass is the largest component of fat‐free mass (FFM) reported to be 0.49 × FFM in human patients.^8^ Determination of FFM therefore may provide an estimate of muscle mass. In addition, morphometric measurements, body condition score (BCS), and body weight (BW) also may provide surrogate markers of muscle mass. A formula to predict FFM in cats based on BW (kg) and various morphometric measurements has been reported (Finch et al., *J Vet Intern Med*. 2010; 24: 1548 [abstract]).

No published eGFR formulas have been developed for veterinary species and such a formula may prove to be a more useful indicator of renal function than serum creatinine concentration alone. Our objectives were to develop an eGFR formula in cats with various levels of renal function based on noninvasive measurements including signalment, morphometric measurements, FFM, and predicted FFM by which to adjust serum creatinine concentration. A second objective was to test the formula in another group of cats.

## 2 MATERIALS AND METHODS

### 2.1 Study population

Client‐owned cats with various levels of renal function were recruited into the study, but cats with evidence of concurrent medical disease such as hyperthyroidism were excluded. Only cats with stable renal function determined by at least 2 repeated serum creatinine concentration measurements within 12 months in nonazotemic cats and within 3 months in azotemic cats before GFR measurement were included in the study. Cats with evidence of acute kidney injury were excluded from the study. The cats were identified from an ongoing cohort study conducted at 2 London‐based first opinion practices (Beaumont Sainsbury Animals' Hospital [BAH], Royal Veterinary College, Camden and People's Dispensary for Sick Animals [PDSA], Bow). The cats in this study were presented for senior cat wellness screening. At each visit, a full medical history was obtained, physical examination performed, urine sample collected by cystocentesis, blood pressure measured by the Doppler technique and blood collected for hematology, biochemistry and total serum thyroxine concentration measurement. This approach allowed cats to be followed longitudinally during which time some develop azotemic CKD and some remained nonazotemic. Cats had no evidence of relevant clinical disease. Informed consent was obtained from the owners and the study was conducted with approval from the Royal Veterinary College's ethics and welfare committee.

### 2.2 Measurement of GFR

Food was withheld for 12 hours before performing the measurements. Glomerular filtration rate was determined by a previously described iohexol clearance method.^9^ Briefly, a bolus dose of iohexol (Omnipaque 300 [647 mg/mL; 300 mg of iodine/mL, GE Healthcare, Wauwatosa, Wisconsin) was administered IV (1 mL/kg). Blood samples were collected at 120, 180, and 240 minutes postinjection. Iohexol concentrations were determined at an external commercial laboratory by a high performance liquid chromatography (HPLC) method (Epsom and St Helier University Hospital NHS trust, Epsom, United Kingdom). Clearance was determined as dose/area under the curve (AUC) where AUC was the area under the plasma concentration vs time curve determined by a 1‐compartment model. A previously validated cat‐specific correction formula for slope‐intercept clearance was applied to correct for the 1‐compartment assumption.^9^ In addition, serum creatinine, urea, albumin, and total protein concentrations were determined from a sample collected at the same time as GFR measurement.

### 2.3 Development of eGFR formula

The following variables were considered for inclusion in a multivariable regression model to predict eGFR: age, sex (categorized as either male neutered or female neutered), breed (categorized as either domestic short hair/long hair or pedigree), urea, albumin, total protein, BW, BCS, predicted muscle mass, various morphometric measurements, FFM, and predicted FFM. Body condition score was determined by a previously validated 9‐point scaling system.^10^ Fat‐free mass was calculated by the equation^11^:

$$\mathrm{FFM}=\text{Total body water}\;\left(\mathrm{TBW}\right)/0.74$$

where TBW was calculated by an 18‐Oxygen (^18^O) dilution method (Finch et al., *J Vet Intern Med*. 2010; 24: 1548 [abstract]). Briefly, baseline blood samples and samples collected after an equilibration period of 2 hours after administration of ^18^O (0.3 g/kg 10% solution H_2_^18^O) were analyzed at an external laboratory (Institute of Child Health, London) by isotope ratio mass spectrometry (IRMS). Dilution space of ^18^O was calculated by the standard equation^12^:

$$\text{Dilution space}\;{\;}^{18}O=\;\frac{\;T\times A}{a}\times \;\frac{\left(\mathrm{\delta a}–\mathrm{\delta t}\right)}{\left(\mathrm{\delta s}–\mathrm{\delta p}\right)}$$

where *T* is the mass of tap water diluent in which *a* is diluted, *A* is dose of ^18^O administered, *a* is portion of dose administered of ^18^O that was retained for mass spectrometer analysis, and δ_a_, δ_t_, δ_s_, and δ_p_ are isotopic enrichment in delta units of the portion of dose administered, tap water diluent, postdose serum sample, and predose serum sample, respectively. Delta units express isotopic enrichment relative to 2 standard waters (standard mean ocean water and standard light arctic precipitate). Total body water then was calculated as:

$${\;}^{18}O\;\text{dilution space}/1.01$$

where 1.01 is the correction factor to correct for 1% over‐exchange with nonaqueous compartments.

Predicted muscle mass was determined by the equation^13^:

$$\text{Predicted muscle mass}=0.468\times {\mathrm{BW}}^{0.99}$$

**Figure 1:**
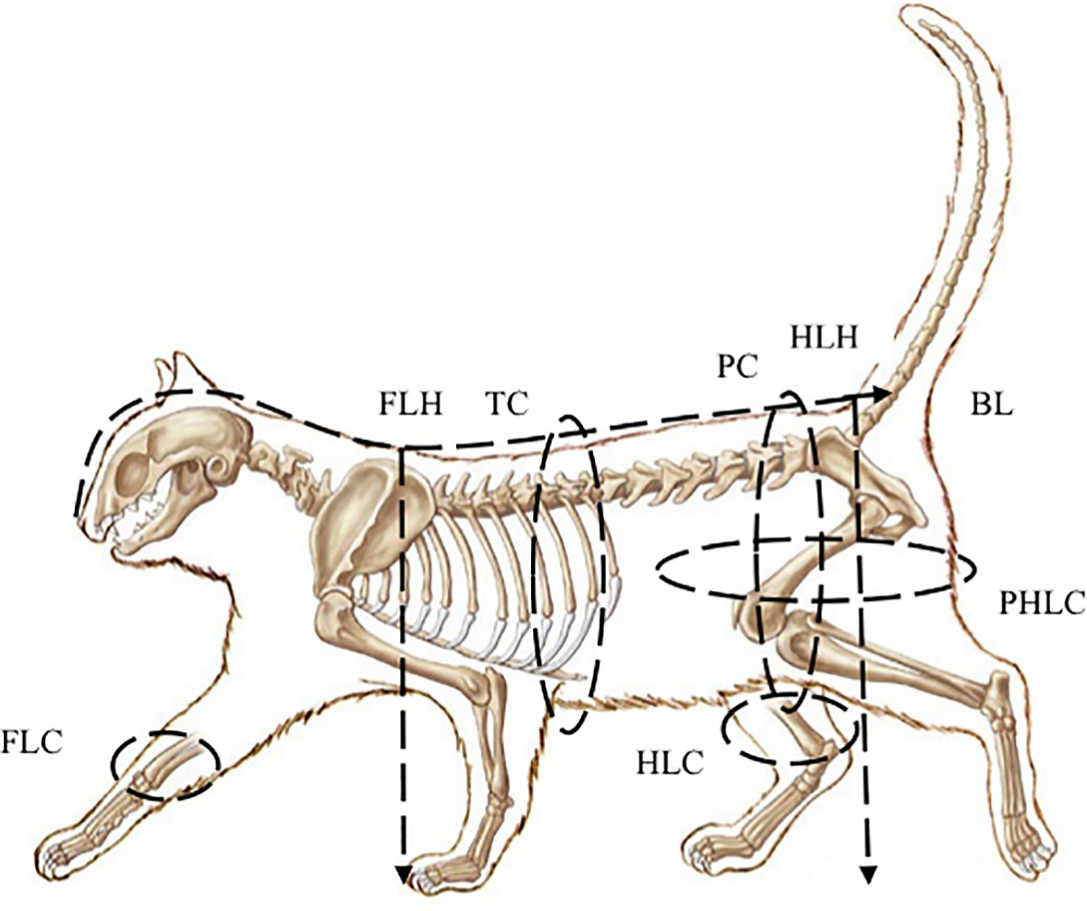
Diagram of a cat illustrating the morphometric measurements recorded. Morphometric measurements were determined using a flexible tape measure and determined to nearest 0.1 cm. BL ‐ body length, FLC ‐ forelimb circumference, HLC ‐ hindlimb circumference, FLH ‐ forelimb height, HLH ‐ hind limb height, PC ‐ pelvic circumference, PHLC ‐ proximal hindlimb circumference, TC ‐ thoracic circumference

Morphometric measurements were performed with a flexible tape measure and recorded to the nearest 0.1 cm. The same investigator performed the measurements in all cats to minimize interobserver variability. Cats stood in a standard position when measurements were obtained. The following morphometric measurements were recorded (Figure 1): left and right forelimb circumference (FLC) measured 3 cm proximal to the carpus, left and right hindlimb circumference (HLC) measured 3 cm proximal to the tarsus, left and right proximal hindlimb circumference (PHLC) measured 1 cm proximal to the patella, thoracic circumference (TC) measured at the level of the xiphoid process, pelvic circumference (PC) measured at the level of the ilium, body length (BL) measured from nose tip to sacrococcygeal joint, forelimb height (FLH) measured from ground to the dorsal border of scapula, and hindlimb height (HLH) measured from ground to the dorsal border of pelvis.

Predicted FFM was calculated by the equation (Finch et al., *J Vet Intern Med*. 2010; 24: 1548 [abstract]):

Predicted FFM = −0.164 + (0.41 × BW) + (0.054 × FLH) + (0.098 × RFLC) ‐ (0.028 × HLH)

where BW is body weight (kg), FLH is forelimb height (cm), RFLC is right forelimb circumference (cm), and HLH is hindlimb height (cm).

Statistical analyses were performed by a statistical software package (SPSS version 17.0). Data were assessed for normality by the Kolmogorov‐Smirnov test and by visual inspection of graphical plots. Data between the population in which the formula was developed and that in which it was tested was compared by the Student's *t* test. Where assumptions of linearity and Gaussian distribution were not met, transformations were performed. Univariable linear regression analysis was performed for each individual variable initially. Variables with a *P* < .2 were entered into a manual forward stepwise linear regression model containing the reciprocal of the serum creatinine concentration to predict eGFR. The reciprocal of the serum creatinine concentration was selected because it displayed a linear relationship to GFR compared to that of serum creatinine concentration, which was nonlinear. Predicted GFR was expressed as mL/min/kg. Model assumptions and performance were evaluated by examining multicollinearity, standardized residuals, Cook's distances, leverage values, and by performing the Durbin‐Watson test. Significance was set at *P* < .05.

### 2.4 Testing of eGFR formula

The derived eGFR formula was subsequently applied to an additional 25 cats in which GFR assessed by iohexol clearance had been determined and performance tested by assessing the coefficient of determination (*R* ^2^). Agreement was assessed by creating Bland‐Altman plots.^14^

## 3 RESULTS

### 3.1 Development of the eGFR formula

Data regarding signalment, renal function, and body composition are included in Table 2. The following variables were significant in the univariable analysis: reciprocal of serum creatinine concentration (*P* < .001), serum urea concentration (*P* < .001), left forelimb circumference (*P* = .019), and right proximal hindlimb circumference (*P* = .047; Table 3).

**Table 2:** Clinical data relating to population of cats in which the eGFR was developed and the population of cats in which the eGFR formula was tested. Continuous variables are presented as mean ± SD. FN ‐ female neutered, MN ‐ male neutered, DSH/DLH ‐ domestic short hair/domestic longhair

	eGFR formula development population (*n* = 55)	eGFR formula testing population (*n* = 25)	*P* value
Age (yrs)	12.8 ± 3.2	12.4 ±3.7	.626
Sex	FN *n* = 27 MN *n* = 28	FN *n* = 13 MN *n* = 12	.830
Breed	DSH/DLH *n* = 40 Pedigree *n* = 15	DSH/DLH *n* = 17 Pedigree *n* = 8	.885
GFR (mL/min/kg)	1.54 ± 0.57	1.87 ± 0.56	.016
Serum creatinine concentration (μmol/L)	154.15 ± 50.55	155.89 ± 52.86	.941
Reciprocal of serum creatinine concentration (μmol/L)	0.007 ± 0.002	0.007 ± 0.002	.925
Serum urea concentration (mmol/L)	12.02 ± 3.80	12.28 ± 4.00	.895
USG	1.046 ± 0.022	1.039 ± 0.021	.200
Body weight (kg)	4.42 ± 1.14	4.20 ± 1.31	.463
Body condition score (1‐9)	6 ± 1	5 ± 1	.228
Fat‐free mass (kg)	3.07 ± 0.62	Not measured	n/a
Predicted fat‐free mass (kg)	3.05 ± 0.59	3.02 ± 0.71	.909
Predicted muscle mass (kg)	2.02 ± 0.51	1.99 ± 0.66	.824
Left forelimb circumference (cm)	8.29 ± 0.94	7.91 ± 0.96	.103
Right forelimb circumference (cm)	8.05 ± 0.91	8.12 ± 0.89	.621
Left hindlimb circumference (cm)	9.22 ± 1.01	8.93 ± 1.12	.242
Right hindlimb circumference (cm)	9.03 ± 1.08	8.82 ± 0.95	.416
Left proximal hindlimb circumference (cm)	24.60 ± 4.04	23.30 ± 2.80	.152
Right proximal hindlimb circumference (cm)	25.95 ± 4.30	23.72 ± 3.36	.026
Thoracic circumference (cm)	40.34 ± 4.93	39.28 ± 4.84	.378
Pelvic circumference (cm)	41.00 ± 7.04	38.48 ± 6.73	.141
Body length (cm)	53.30 ± 5.14	53.28 ± 2.71	.983
Forelimb height (cm)	26.70 ± 3.13	36.93 ± 3.04	.764
Hindlimb height (cm)	29.61 ± 3.29	28.14 ± 3.04	.062

**Table 3:** Univariable analysis of predictors of GFR used to develop an eGFR formula. Univariable analysis was performed in 55 cats. Significant variables are highlighted in bold font

Variable	*R* ^2^	*P* value
Age	0.046	.115
Sex	0.059	.075
Breed	0.038	.154
**Reciprocal of serum creatinine concentration**	**0.437**	**<.001**
**Serum urea concentration**	**0.258**	**<.001**
Serum albumin concentration	0.004	.631
Serum total protein concentration	0.017	.339
Body weight	0.053	.090
Body condition score	0.002	.749
Predicted muscle mass	0.037	.196
Fat‐free mass	0.023	.318
Predicted fat‐free mass	0.041	.151
**Left forelimb circumference**	**0.099**	**.019**
Right forelimb circumference	0.010	.472
Left hindlimb circumference	0.006	.560
Right hindlimb circumference	0.010	.464
Left proximal hindlimb circumference	0.053	.090
**Right proximal hindlimb circumference**	**0.074**	**.047**
Thoracic circumference	0.018	.330
Pelvic circumference	0.054	.089
Body length	0.048	.107
Forelimb height	0.005	.605
Hindlimb height	0.000	.970

Except for the reciprocal of serum creatinine concentration, variables describing signalment and serum parameters (listed in Table 3) were not predictive of GFR in the final multivariable regression model. Fat‐free mass estimated by the cat prediction formula also was not predictive of GFR. Only a single morphometric measurement remained significant in the model to predict GFR with the reciprocal of serum creatinine concentration. Three cats were excluded from the final regression model because they did not meet the model assumptions. The final model used to develop the formula therefore included 52 cats. The derived eGFR formula was:

eGFR = 0.408 + (243.11 × creatinine^−1^ [μmol/L]) ‐ (0.014 × PC [cm])

The model *R* ^2^ was 0.67 (*P* < .001) and explained additional variation over the reciprocal of serum creatinine concentration alone (*R* ^2^ = 0.64, *P* < .001).

### 3.2 Testing of the eGFR formula

**Figure 2:**
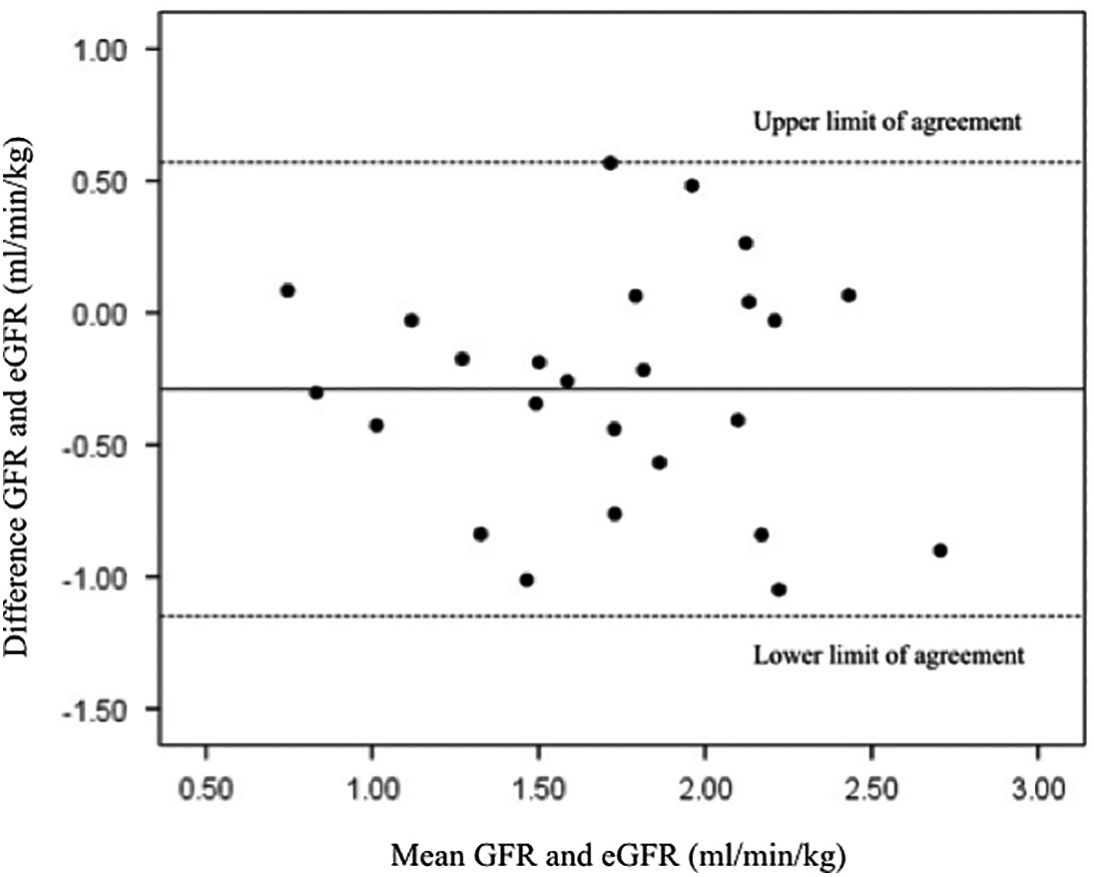
Bland‐Altman agreement plot showing agreement between GFR (determined by iohexol clearance) and estimated GFR (eGFR). Bold line represents bias (mean difference between GFR and eGFR) and dashed lines represent upper and lower limits of agreement (mean difference between GFR and eGFR ±2 SD). The bias indicated eGFR underestimated GFR and limits of agreement were wide. Therefore, agreement was considered poor

**Figure 3:**
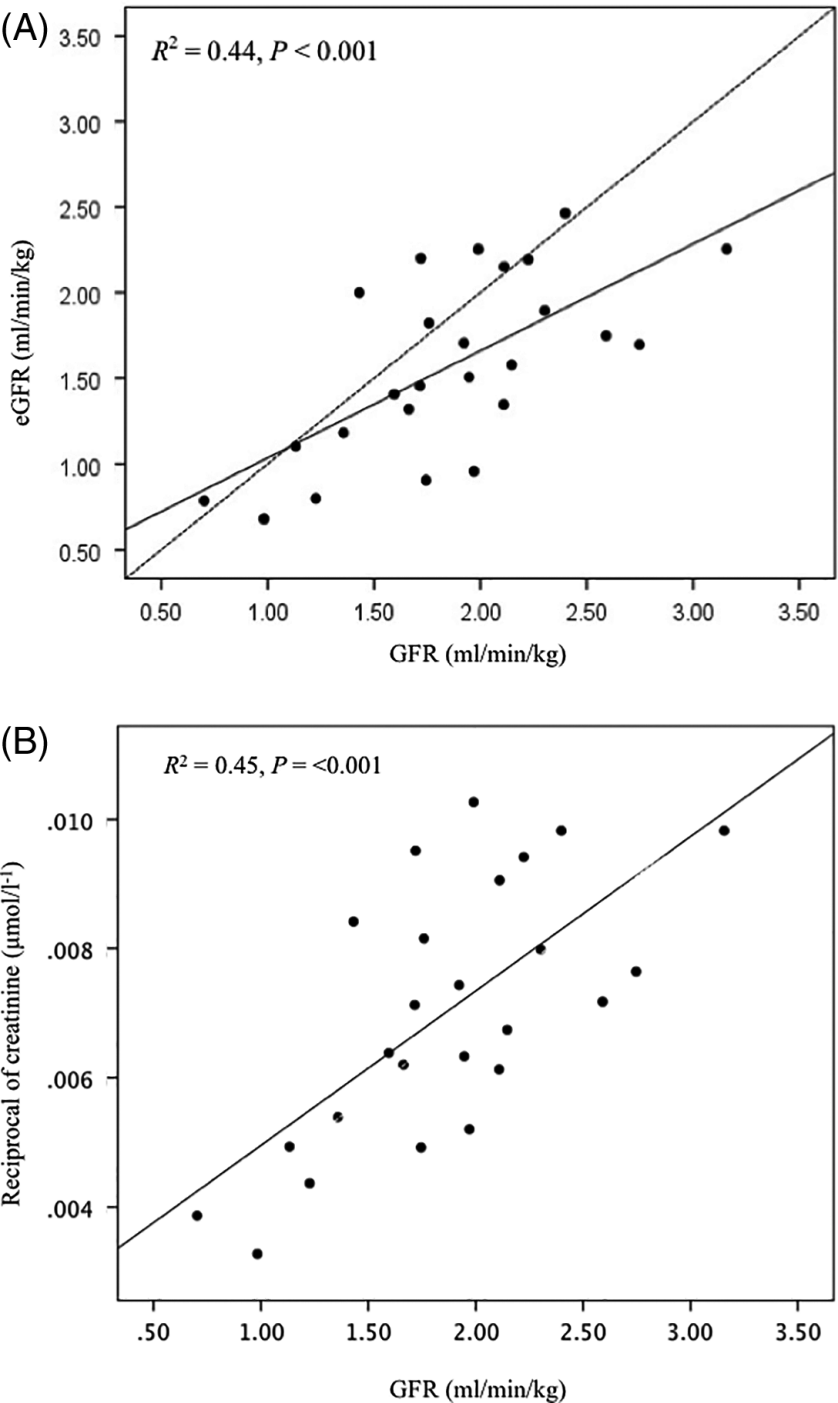
(A) Relationship between GFR (determined by iohexol clearance) and estimated GFR (eGFR). eGFR underestimated GFR. Bold line is regression line for GFR and eGFR and dashed line is line of equality.(B) relationship between GFR (determined by iohexol clearance) and the reciprocal of serum creatinine concentration. Bold line is regression line for GFR and the reciprocal of serum creatinine concentration

Data regarding signalment, BW, and renal function are included in Table 2. The mean ± SD PC was not significantly different between the development and test population (*P* = .141, 41.0 ± 7.1, and 38.5 ± 6.7 cm, respectively). Agreement between GFR and eGFR was considered poor based on the wide limits of agreement (−1.15‐0.57 mL/min/kg). The negative bias (−0.29 mL/min/kg) also indicated that eGFR underestimated GFR (Figure 2). The mean percent error in 25 cats in which the eGFR formula for cats was tested was −13.6%. The relationship between GFR and eGFR and GFR and the reciprocal of serum creatinine concentration is presented in Figure 3. The eGFR formula showed no advantage over the reciprocal of serum creatinine concentration in its relationship with GFR (eGFR *R* ^2^ = 0.44, *P* < .001 vs reciprocal of serum creatinine concentration *R* ^2^ = 0.45, *P* < .001).

## 4 DISCUSSION

In our study, the morphometric measurement, PC, was the only significant measurement considered to be a surrogate marker of muscle mass for correcting the reciprocal of serum creatinine concentration to predict GFR in a multivariable regression model. This eGFR formula had a slightly stronger relationship to GFR (determined by iohexol clearance) than the reciprocal of serum creatinine concentration in cats in which the formula was developed (*R* ^2^ = 0.67 vs *R* ^2^ = 0.64). However, when the formula was tested in an additional 25 cats, it was not found to offer any improvement in predicting GFR than the reciprocal of the serum creatinine concentration alone (*R* ^2^ = 0.44 vs *R* ^2^ = 0.45). Agreement of the eGFR formula with GFR was considered poor based on the wide limits of agreement (−1.15‐0.57 mL/min/kg) and negative bias (−0.29 mL/min/kg).

Clinical concerns regarding accuracy of the eGFR formulas used in humans in all patient populations along with the sensitivity and specificity of the formulas have been expressed. The precision and accuracy of the formulas are questionable, particularly in elderly patients, patients with extremes of muscle mass, and in patients with unstable renal function. Furthermore, the earlier formulas^3^, ^4^ may underestimate true GFR leading to misclassification of some patients as having CKD. In human patients, the correlation coefficient (*r*) between GFR and eGFR predicted by the Cockcroft‐Gault formula was 0.83 and mean percentage error (expressed as a percentage of true GFR) was 35% in 95% of patients.^3^ In the study that developed the MDRD formula, the *R* ^2^ for true and predicted GFR was 0.9 and percentage error was 28.4% in 90% of the population.^4^ The main difference between the 2 populations was that the MDRD formula included a more diverse population and studied additional factors such as age, sex, and ethnicity and included only patients with CKD. The Cockcroft‐Gault population did not include such a varied population, was only developed in hospitalized males, and data regarding whether any patients had CKD were not included. When the abbreviated MDRD formula was applied to CKD patients, the *R* ^2^ was 0.79, but in normal patients the *R* ^2^ was only 0.19.^15^ Furthermore, mean percentage error was −6.2% in CKD patients and −29% in healthy patients.^15^ The most recently developed prediction formula for GFR in human patients is the CKD‐EPI formula. Because this formula was developed in patients with decreased and normal renal function, it is considered appropriate for both populations. It is reported that 84.1% of patients had GFR estimates within 30% of true GFR, by this formula.^5^ In our study, 68% of cats had an eGFR within 30% of GFR. When the relationship between serum creatinine concentration and GFR was explored in the study in which the MDRD equation was developed, the *R* ^2^ was 0.8 (vs 0.9 for GFR and eGFR),^4^ suggesting these formulas do offer some advantage over the use of serum creatinine concentration alone. However, it is clear from the mean percent errors that the formulas are not particularly precise predictors of GFR in humans. Mean percent error in 25 cats in which the eGFR formula for cats was tested was −13.6%, which, interestingly, is considerably lower than that obtained in some of the studies in humans. An important conclusion from studies in humans that derived prediction equations for GFR^4^, ^15^ is that the population in which formulas are developed should represent the target population. Our study addressed this concern by inclusion of normal healthy cats and cats with decreased renal function in development and testing of the equation. It is disappointing that the eGFR formula for cats did not appear to offer any advantage over measurement of the reciprocal of serum creatinine concentration to predict GFR, which may reflect the small number of cats included in the development of the formula. Until a more accurate formula can be developed in a larger population of cats, biomarkers such as serum creatinine or symmetric dimethylarginine concentrations remain the best surrogate markers of GFR in cats.

The primary source of creatinine generation is muscle mass. This factor is addressed in human patients by including coefficients in GFR prediction equations for factors affecting muscle mass such as age, sex, and race. It is likely that there are important differences in creatinine generation in cats. It has been reported that serum creatinine concentration is higher in Birman cats^16^ although whether this observation relates to lower GFR or increased creatinine generation is unclear. Furthermore, chronic disease such as CKD can decrease muscle mass, and therefore cats with chronic disease with the same serum creatinine concentration as healthy cats will have lower GFR if measured. This leads to a circular argument in which serum creatinine concentration or adjusted serum creatinine concentration is used to predict GFR and determine if a patient has normal or abnormal renal function, but CKD itself will affect serum creatinine concentration and hence also affect accurate estimation of renal function by an eGFR formula. With this is mind, any eGFR formula that is validated for cats should serve only as a screening test. In addition, a useful eGFR formula should not only serve as a useful screening test but also provide reliability and accuracy for monitoring progression of CKD.

When using eGFR formulas it is important to ensure that identical methods of analysis and the same laboratory are employed as those used for deriving the formula. Differences in determination of serum creatinine concentration, for example, if a method that detected noncreatinine chromogens was used, would lead to errors in predicted GFR. In studies of humans, differences in creatinine assays at different clinical laboratories can cause errors in GFR estimation as high as 20%.^17^ Differences in serum creatinine concentrations determined at different veterinary practices have been reported, making this factor a relevant consideration in cats as well.^18^ Additionally, the same units of measurement of creatinine must be required. The current formula was derived by standard international (SI) units (μmol/L) and creatinine concentrations reported in mg/dl would require conversion to μmol/L before applying the formula.

The formula used to estimate FFM in our study does not predict muscle mass. Skeletal muscle mass is the largest component of FFM (reported to be 0.49 × FFM in human patients).^8^ No ratio has been reported for cats. A method to determine skeletal muscle mass may be important in cats because of its relationship with serum creatinine concentration. In our study, it was not possible to measure muscle mass directly and therefore predicted FFM was determined. The formula for FFM in cats has been shown to provide good prediction of true FFM determined from total body water and the hydration constant in cats (Finch et al., *J Vet Intern Med*. 2010; 24: 1548 [abstract]). In pediatric patients, correcting serum creatinine concentration for body surface area or body mass index was not found to improve the accuracy of GFR prediction.^19^ Further studies to explore the relationships among muscle mass, serum creatinine concentration, and GFR are required in cats. Larger studies performed in a more varied population of cats to that included in our study may be needed before a reliable eGFR formula can be recommended. Doing so may involve direct measurement of muscle mass rather than FFM, although this may be difficult to achieve in clinical patients, and other factors such as sex, age, breed and disease state may influence GFR and serum creatinine concentration.

Development of an eGFR formula for cats to correct creatinine for body composition in our study did not provide a reliable estimate of GFR in cats, and therefore its routine use cannot be recommended. Moreover, the formula does not appear to improve the accuracy of predicting GFR over serum creatinine concentration. Therefore, determination of GFR will remain important in the early identification and accurate assessment of the stage of CKD.

## CONFLICT OF INTEREST DECLARATION

Authors declare no conflict of interest.

## References

[1] Finch N . Measurement of glomerular filtration rate in cats: methods and advantages over routine markers of renal function. J Feline Med Surg. 2014;32:1970‐1976. 10.1177/1098612X14545274 PMC11185244 25146661

[2] Glassock RJ , Winearls C . Ageing and the glomerular filtration rate: truths and consequences. Trans Am Clin Climatol Assoc. 2009;120:419‐428. 19768194 PMC2744545

[3] Cockcroft DW , Gault MH . Prediction of creatinine clearance from serum creatinine. Nephron. 1976;16:31‐41. 1244564 10.1159/000180580

[4] Levey AS , Bosch JP , Lewis JB , et al. A more accurate method to estimate glomerular filtration rate from serum creatinine: a new prediction equation. Ann Intern Med. 1999;130:461. 10075613 10.7326/0003-4819-130-6-199903160-00002

[5] Levey AS , Stevens LA , Schmid CH , et al. A new equation to estimate glomerular filtration rate. Ann Intern Med. 2009;150:604‐612. 19414839 10.7326/0003-4819-150-9-200905050-00006 PMC2763564

[6] James MT , Hemmelgarn BR , Tonelli M . Early recognition and prevention of chronic kidney disease. Lancet. 2010;375:1296‐1309. 20382326 10.1016/S0140-6736(09)62004-3

[7] Munday HS . Assessment of body composition in cats and dogs. Int J Obes Relat Metab Disord. 1994;18(Suppl 1):S14‐S21. 8087160

[8] Heymsfield SB , Gallagher D , Visser M , et al. Measurement of skeletal muscle: laboratory and epidemiological methods. J Gerontol. 1995;50:23‐29. 10.1093/gerona/50a.special_issue.23 7493213

[9] Finch NC , Syme HM , Elliott J , et al. Glomerular filtration rate estimation by use of a correction formula for slope‐intercept plasma iohexol clearance in cats. Am J Vet Res. 2011;72:1652‐1659. 22126694 10.2460/ajvr.72.12.1652

[10] Laflamme D . Development and validation of a body condition score system for cats: a clinical tool. Feline Pract. 1997;25:13‐18.

[11] Elliott DA . Evaluation of multifrequency bioelectrical impedence analysis for the assessment of body composition in cats and dogs. California, US: University of California, Davis; 2001.

[12] Wells JC , Ritz P , Davies PS , et al. Factors affecting the ^2^H to ^18^O dilution space ratio in infants. Pediatr Res. 1998;43:467‐471. 9544999 10.1203/00006450-199804000-00005

[13] Wang Z , Deurenberg P , Wang W , Pietrobelli A , Baumgartner RN , Heymsfield SB . Hydration of fat‐free body mass: review and critique of a classic body‐composition constant. Am J Clin Nutr. 1999;69:833‐841. 10232621 10.1093/ajcn/69.5.833

[14] Bland JM , Altman DG . Statistical methods for assessing agreement between two methods of clinical measurement. Lancet. 1986;1:307‐310. 2868172

[15] Rule AD , Larson TS , Bergstralh EJ , Slezak JM , Jacobsen SJ , Cosio FG . Using serum creatinine to estimate glomerular filtration rate: accuracy in good health and in chronic kidney disease. Ann Intern Med. 2004;141:929‐937. 15611490 10.7326/0003-4819-141-12-200412210-00009

[16] Gunn‐Moore DA , Dodkin SJ , Sparkes AH . An unexpectedly high prevalence of azotaemia in Birman cats. J Feline Med Surg. 2002;4:165‐166.1235451710.1053/jfms.2002.0175

[17] Levey AS , Coresh J , Balk E , et al. National kidney foundation practice guidelines for chronic kidney disease: evaluation, classification, and stratification. Ann Intern Med. 2003;139:137‐147. 12859163 10.7326/0003-4819-139-2-200307150-00013

[18] Braun JP , Cabe E , Geffre A , Lefebvre HP , Trumel C . Comparison of plasma creatinine values measured by different veterinary practices. Vet Rec. 2008;162:215‐216. 18281630 10.1136/vr.162.7.215

[19] Hjorth L , Wiebe T , Karpman D . Correct evaluation of renal glomerular filtration rate requires clearance assays. Pediatr Nephrol. 2002;17:847‐851. 12376815 10.1007/s00467-002-0913-3

